# A PTSD symptoms trajectory mediates between exposure levels and emotional support in police responders to 9/11: a growth curve analysis

**DOI:** 10.1186/s12888-016-0907-5

**Published:** 2016-07-04

**Authors:** Ralf Schwarzer, James E. Cone, Jiehui Li, Rosemarie M. Bowler

**Affiliations:** Institute for Positive Psychology and Education, Australian Catholic University, Sydney, Australia; SWPS University of Social Sciences and Humanities, Wroclaw, Poland; World Trade Center Health Registry, Division of Epidemiology, New York City Department of Health and Mental Hygiene, New York, NY USA; Department of Psychology, San Francisco State University, San Francisco, CA USA

**Keywords:** Social support, Trauma, Posttraumatic stress disorder, Stress, Symptoms, PTSD

## Abstract

**Background:**

Exposure to the terrorist attack on the World Trade Center (WTC) on 9/11/2001 resulted in continuing stress experience manifested as Posttraumatic Stress Disorder (PTSD) Symptoms in a minority of the police responders. The WTC Health Registry has followed up a large number of individuals, including police officers, at three waves of data collection from 2003 to 2011. This analysis examines the relationship between initial exposure levels, long-term PTSD symptoms, and subsequent emotional support among police responders.

**Methods:**

The study population included police responders who had reported their 9/11 exposure levels at Wave 1 (2003/4), provided three waves of data on PTSD symptoms using the 17-item PCL scale, and rated their received emotional support at Wave 3 (*N* = 2,204, 1,908 men, 296 women, mean age: 38 years at exposure). A second-order growth curve reflected a PTSD symptom trajectory which was embedded in a structural equation model, with exposure level specified as an exogenous predictor, and emotional support specified as an endogenous outcome.

**Results:**

Exposure had a main effect on mean symptom levels (intercept) across three waves but it made no difference in changes in symptoms (slope), and no difference in emotional support. The symptom trajectory, on the other hand, had an effect on emotional support. Its intercept and slope were both related to support, indicating that changes in symptoms affected later emotional support.

**Conclusions:**

Initial trauma exposure levels can have a long-term effect on mean symptom levels. Emotional support is lower in police responders when PTSD symptoms persist over seven years, but becomes higher when reduction in symptoms occurs.

## Background

The September 11, 2001 terrorist attacks on the New York World Trade Center (WTC) when two airplanes were piloted into the twin towers, caused a considerable amount of injuries and almost 2,800 deaths [1–5]. First responders rushed to the scene within minutes and hours of the attack, witnessing victims who jumped from the burning and collapsing buildings. Police officers guarded the perimeter of the disaster site, assisted survivors, and participated in the search for bodies and body parts. The response and rescue to survivors and the search for body parts caused severe stress to those engaged in this process. This resulted in a mental health challenge and onset of posttraumatic stress disorder (PTSD) and/or anxiety and depression for some of the officers. On the other hand, a large number of the officers did not develop long-term mental health conditions but were reported to be resilient [6–8].

The World Trade Center Health Registry (WTCHR) is following a cohort of 71,437 individuals who were directly involved by performing disaster-related rescue tasks on the day of attack, or by living, working or attending school in the lower Manhattan district of New York City, very near the destroyed WTC Towers 1 and 2. The aims of the Registry are to examine the long-term mental and physical health effects of the event, disseminate research findings and recommendations to participants and to others who were exposed, and to provide information about resources and services to the public and to the scientific community. Between September 2003 and November 2004, Wave 1 baseline assessments took place, informed consent was given and enrollees completed a computer-assisted telephone interview (94.5 %) or in-person (5.5 %) interview regarding demographic data, exposure levels, mental health and physical symptoms, as well as medical conditions [7]. About two to three years after the disaster, some of the 4,017 police officers (582 women, 3,435 men) reported to have experienced continuing distress, and they met criteria for PTSD [9] in the Wave 1 survey by the WTCHR.

At Wave 2, conducted from November 2006 to December 2007, self-reported symptoms and medically diagnosed illnesses were assessed again, and questions on social integration were incorporated. At the Wave 3 data collection, conducted in 2011 and 2012, emotional support questions were also included.

Various research studies on the first responders have reported mental health conditions, including posttraumatic stress disorder (PTSD), anxiety, depression, panic disorder, or anger associated with rescue and recovery work in the context of the twin tower disaster [1, 2, 4–6]. In the Registry’s Wave 2 study [7] five to six years after the attacks, the prevalence of PTSD among police officers was 16.5 %, higher than two to three years after the event. The incidence of PTSD among officers enrolled in the Registry increased from 2.5 % two years after the attacks to 6.3 % six years after the attacks [10]; ten to eleven years after the attacks, 11 % of the officers who participated in all three waves, had probable PTSD according to DSM-IV criteria, and the annual PTSD incidence peaked in the fourth year after the attacks and then stabilized [10]. Additionally, police officers’ comorbidity of PTSD, depression, and anxiety was examined [11]; of 243 enrollees with probable PTSD only 21.8 % did not have comorbid conditions, whereas 24.7 % of this PTSD group also had diagnosed depression, and 5.8 % had a diagnosis of anxiety. When investigating probable PTSD of other 9/11 victims, police officers were found to be more resilient than other responders to the disaster such as construction workers [12]. A more detailed account of the Wave 3 research results was reported previously [8].

A follow-up of these results in the present analysis, in all three waves of data collection, examines exposure levels as a distal predictor of PTSD symptoms, and individual differences in emotional support as a consequence of symptom levels. Individual differences in exposure levels are the most obvious antecedents of subsequent stress responses, including PTSD. While working as first responders after the WTCattacks in the rescue and recovery efforts and later cleanup of the site, police officers had to recover human body parts, may have witnessed the airplanes hitting the towers and witnessed the buildings collapsing, people running away from clouds of smoke, and witnessed people injured or killed, or and people jumping and falling from the towers [4, 13]. Such experiences along with risk of personal injury may be a major cause of their development of PTSD [14].

Social support pertains to the quality and function of social relationships, which can be of the more instrumental, emotional, or informational type. It can refer to the perceived availability of help or to the assistance reported as being received. In research, social support has been specified as an independent, buffering, or dependent variable [15–17]. In an earlier reanalysis of the first two waves of the WTCHR Registry data collection of the police officers, the buffering role of social integration had been examined [18]. In contrast to social support, social integration pertains to the quantity and structure of social relationships, including the social interaction frequency as well as the size and density of networks. The present reanalysis takes a different view on the three-wave data set with a focus on emotional support levels as an outcome. Due to its measurement at Wave 3, emotional support is specified as a dependent variable, assuming that chronic symptoms, about one decade after the traumatic events, may have decreased emotional support in those who suffered the most. Thus, psychological determinants of individual differences in received emotional support at the end of the observed time window are of interest here.

### Aims

This reanalysis of the WTCHR three-wave data focused on the relationship between initial exposure levels, PTSD symptoms, and emotional support. In the center of the analysis is a growth curve modeling approach to yield a stress symptom trajectory comprising the seven-year period from Wave 1 to Wave 3 of data collection. Within a structural equation model, the intercept and slope of this symptom trajectory are then specified between initial exposure levels and subsequent individual differences in received emotional support. The main research questions are: Does initial exposure level affect the symptom trajectory in the following seven years? Does symptom history from 2003/4 to 2010/11 correlate with differences in received emotional support reported in the Wave 3 data.

## Methods

### Participants and procedures

The study sample comprises police first responders to the 9/11 twin tower attacks. Officers were enrolled in the WTC Health Registry cohort. Informed consent was given to New York City (NYC) Department of Health and Mental Hygiene (DOHMH). All details concerning enrollment and follow-up surveys have been described elsewhere [1, 8, 13]. Wave 3 assessment was conducted by the Registry from June 2011 to March 2012 to collect mental and physical health data through web surveys (45 %), paper surveys (41 %), and phone interviews (14 %) [19]. To be included in this analysis, officers must have worked at least one shift between September 11, 2001, and June 30, 2002 at the towers or related areas, or must have been involved in the transportation of the debris between the towers and barges after the attack. In addition, this study is limited to those first responders without PTSD prior to the 9/11 disaster, and who completed all items of the PTSD Checklist (PCL) at each of the Waves 1, 2 and 3 [20]. Inclusion criteria were met by 2,204 officers (1,908 men, 296 women). Average age at exposure (in 2001) was 38.01 years (*SD* = 7.26, range = 19–68 years). Further details on demographics, including response rates and attrition analysis, are reported by Bowler et al. [7] and by Cone et al. [8]. The Centers for Disease Control and Prevention (CDC) and New York City (NYC) Department of Health and Mental Hygiene (DOHMH) institutional review boards approved the Registry protocol.

### Measures

*Stress response* was assessed at all three waves of data collection by symptoms indicative of probable posttraumatic stress disorder (PTSD) using the stressor-specific PTSD Checklist (PCL–Civilian Version), a 17- item self-report instrument [20] based on the Diagnostic and Statistical Manual of Mental Disorders (DSM-IV) criteria [21] and linked to the specific traumatic exposure (“the events of September 11, 2001”). The instructions for their response were asking: “How much have you been bothered by the following problems in the last 30 days?” and included repeated, disturbing memories, thoughts, or images of the events of 9/11. Answers were made on 5-point scales ranging from “not at all” to “extremely”. Cronbach’s alphas were .92 at Wave 1, .95 at Wave 2, and .94 at Wave 3. Possible scores ranged from 17 to 85.

*Exposure levels* were assessed by the sum score of five events that the participants reported witnessing, collected at Wave 1. The interviewers asked participants: “On September 11th, 2001, did you personally witness any of the following?” followed by five scenarios: an airplane hitting the WTC, buildings collapsing, people running away from a cloud of smoke, witnessing anyone who was injured or killed, or witnessing people falling or jumping from the WTC towers. Answers were yes/no, and possible scores ranged from 0 to 5. Cronbach’s alpha was .78. Exposure levels were chosen to serve as an independent variable in the growth curve model.

*Emotional support* was measured by the mean score of three items collected at Wave 3 that were selected from the Modified Social Support Survey (MSSS; [22]). The items asked whether someone was available when needed (a) to have a good time with, (b) to hug them, and (c) to understand their problem. Each item was scored on a 5-point scale: 0 for “none of time,” 1 for “a little,” 2 for “some,” 3 for “most” or 4 for “all of the time”. Cronbach’s alpha was .89. The emotional support scores ranged from 0 to 4.

### Statistical analyses

All analyses were performed with SPSS 23 and MPLUS 7.3. To examine changes in PTSD symptom levels across the time span of seven years for exposure subgroups of police responders, a repeated measures analysis of variance was computed, using self-reported retrospective exposure levels measured at Wave 1 as an independent variable. A structural equation model was included which specified a latent emotional support variable based on three items as indicators at Wave 3. This endogenous construct was predicted by the intercept and slope of the stress symptom trajectory across all three waves. This trajectory was modeled by a second-order growth curve, based on Waves 1, 2, and 3 latent PTSD symptom variables. The latter were indicated by four parcels each, derived from the 17 PCL items at each point in time. Parceling is a common way of selecting latent variable indicators at a higher level than the item-level, if the instrument has many items (here 17 items). This appeared useful because the PCL scale is unidimensional, and the all-item-parcel approach was chosen by randomly selecting from the 17 items new sets of 4, 4, 4, and 5 items to make up the four parcels that serve as the four indicators for each of the three latent variables [23].

Recollected exposure levels, assessed at Wave 1, served as an exogenous factor, specified to affect intercept and slope of the stress symptom trajectory. Model fit was evaluated in terms of the comparative fit index (CFI) and the standardized root mean square residual (SRMR). Missing values (1.3 %) were accounted for by the full information maximum likelihood procedure.

## Results

Preliminary descriptive analyses yielded overall a relatively low mean level of exposure, a high mean level of emotional support, and moderate levels of symptoms. However, previous research on the same sample, using symptom cut-off scores, has pointed to a substantial number of PTSD cases [8]. Table 1 provides an overview of descriptive statistics, and Table 2 provides the correlation matrix.

**Table 1:** Descriptive statistics of study variables

Variable	Mean	*SD*	alpha	Items	Range	*n*
Exposure	1.63	1.62	.78	5	0–5	2,197
Social Support	3.06	1.01	.89	3	0–4	2,184
PCL at Wave 1	25.23	9.39	.92	17	17–85	2,204
PCL at Wave 2	29.47	12.71	.94	17	17–85	2,204
PCL at Wave 3	28.69	12.72	.95	17	17–85	2,204

*Note. PCL* PTSD checklist of stress symptoms [20]

**Table 2:** Correlations of study variables

	Exposure	Support	PCL Wave 1	PCL Wave 2
Exposure Level	1.00			
Social Support	−0.04*	1.00		
PCL Wave 1	0.21**	−0.28**	1.00	
PCL Wave 2	0.19**	−0.31**	0.65**	1.00
PCL Wave 3	0.17**	−0.38**	0.58**	0.72**

*Note. PCL* PTSD checklist of stress symptoms [20]

*P < 0.05; **P < 0.001

**Fig. 1:**
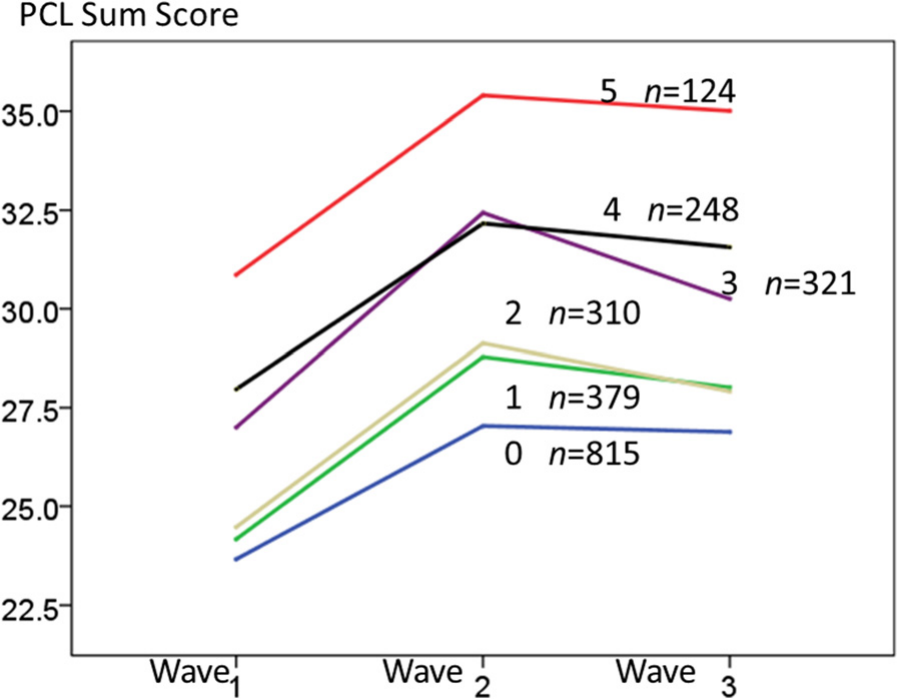
Self-reported retrospective exposure levels (0–5), measured at Wave 1, remain continuously related to PTSD symptom levels across three waves of data collection, 2003–2011. Note. *N* = 2,197. Stress response measured by the PCL checklist of PTSD symptoms [20]. Quadratic interaction between time and exposure (*p* = .006)

Correlations indicate that exposure was positively related to symptoms over time but not to emotional support (*r* = −0.04, *p* = 0.05). Symptoms were negatively related to emotional support (Wave 3 cross-sectional *r* = −0.38, *p* < 0.001). Figure 1 displays the symptom mean levels (PCL sum scores) for six subgroups of participants, ranging from 0 (no exposure) to 5 (high exposure) across three waves. There was a linear main effect of exposure, *F*(1,2191) = 187.99, *p* < 0.001, and a quadratic interaction between time and exposure, *F*(5,2191) = 3.25, *p* = 0.006. Controlling for age and sex did not change this curvilinear pattern. Mean symptom levels have peaked at Wave 2 (Fig. 1).

**Fig. 2:**
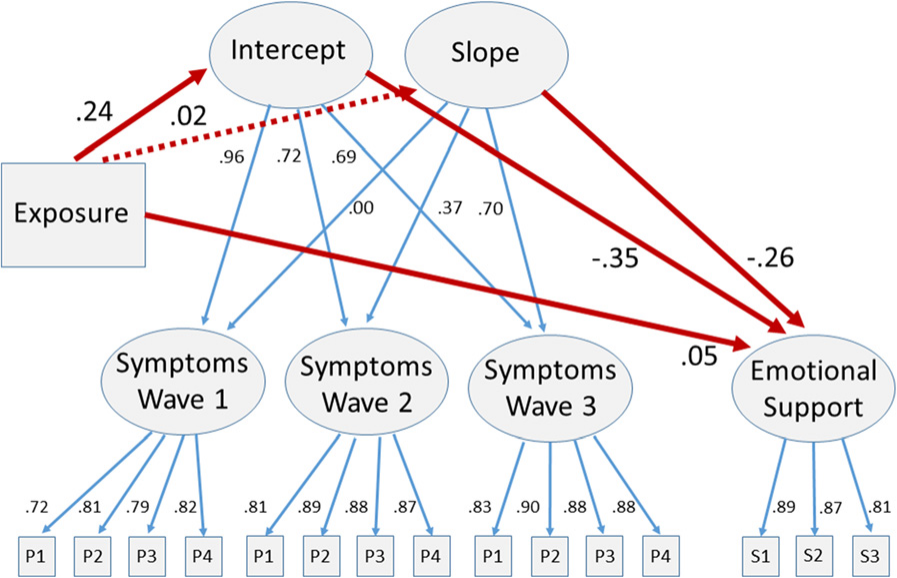
Structural equation model including a second-order linear growth curve model for PTSD symptoms. Exposure is associated with levels of symptoms (intercept) but not with change in symptoms (slope). Levels of symptoms (intercept) affects emotional support at Wave 3, and changes in symptoms (slope) affect support: the steeper the increase in symptoms, the less support will be received (or: the steeper the decrease of symptoms, the more support will follow). Note: Symptoms measured by the PCL checklist of PTSD symptoms [20]. P1, P2, P3, P4 = parcels from 17 item PCL. S1, S2, S3 = Support items. All coefficients are standardized parameter estimates based on maximum likelihood. Non-significant (*P* > 0.05) coefficient is indicated by a dash line

A second-order growth curve model was specified to reflect the trajectory of stress symptoms over seven years. Latent symptom variables were generated at each wave of data collection and modeled as a growth curve. The intercept and slope were then related to Wave 1 recollected exposure levels as an exogenous factor as well as to Wave 3 emotional support as an endogenous latent variable. The model fit to the data was satisfactory with *χ*^2^ (107) = 1,249, *p* < 0.001, CFI =0.94 and SRMR =0.048 (Fig. 2).

Exposure levels were associated with the stress symptom intercept (*ß* = .24, *p* < 0.001), replicating the result of the previous analysis, which means that the more the police were exposed to traumatic events, the more they reported stress symptoms throughout the subsequent time period (Fig. 2). Later changes in symptom levels, however, were not predicted by initial exposure, as indicated by the lack of an association with the slope (*ß* =0.02 *p* > 0.05).

Moreover, the stress symptom trajectory was specified to predict individual differences in emotional support, as reflected by a latent variable at Wave 3. Both intercept (*ß* = −0.35, *p* < 0.001), and slope (*ß* = −0.26, *p* < 0.001) were negatively associated with emotional support. The higher the long-term symptom levels, the lower the later assessed emotional support (intercept effect). Moreover, the steeper the symptoms increased, the lower the support received and vice versa, the steeper the symptoms reduced, the higher the support obtained (slope effect). Of the latent emotional support variance, 18 % was accounted for by the stress symptom trajectory. The indirect effect from Wave 1 exposure levels via the PTSD symptoms trajectory to Wave 3 emotional support was *ß* = −0.09, *p* < 0.001. Compared to the direct effect of *ß* = 0.05, *p* = 0.03, a non-significant total effect of *ß* = -0.04, *p* = 0.10 remained after mediation, underscoring the indirect effects.

## Discussion

This reanalysis of the Registry three-wave database has examined a possible mediation chain, leading from self-reported trauma exposure levels at Wave 1 via a PTSD symptom trajectory to emotional support at Wave 3, 8−9 years later. It was confirmed in this sample of 2,204 police officers that the higher the exposure level, the more stress responses followed over an extended time period [18]. The approach chosen here was to model the longitudinal PTSD symptoms as one latent trajectory comprising three waves of data collection. Exposure levels were associated with the intercept of this trajectory but not with its slope. This means that changes in symptom levels were not affected by initial traumatic exposure but probably by a host of concurrent factors while coping with the aftermath of the event.

Although emotional social support certainly is a relevant factor throughout coping processes, in the present data it is specified as an outcome at the endpoint of the observation period, assuming that long-term symptom experience may affect social interactions. Both intercept and slope of the symptom trajectory were negatively associated with later emotional support, indicating that more symptoms were associated with less support, and that the direction of change also affected support. When police officers reported an increase of PTSD symptomatology over the seven-year period, they found themselves at Wave 3 with less emotional support and vice versa, when they improved over time they also reported more emotional support later on. Cause and effect cannot be determined or disentangled here, although there is temporal precedence of symptom levels suggesting emotional support as an outcome. Change of emotional support could not be determined because the Wave 3 support measures were not administered at earlier waves. This might be possible in analysing the Wave 4 survey currently being readied by the WTCHR for analysis.

In previous research, social support has been shown to be a consistent protective factor for individuals with high distress [15]. Having little or no support was highly associated with the onset and chronicity of the probable PTSD in the same police sample [8]. In a study using the same exposed population but also comprising other workers and survivors, it was found that enrollees with less support were more likely to report unmet needs for mental health care, whereas those with a prior mental health condition made more use of mental health services [24]. Our findings in this report further underscore the potential role of emotional support in reducing distress although the underlying mechanisms need to be further examined in future studies that include several synchronous assessments of independent and dependent variables.

A limitation of the present data lies in their self-report nature and in the lack of additional measurement points in time. All variables were assessed via interviews or questionnaires, and only the subsample of 2,204 officers who completed all PTSD items over the 3 waves, including Wave 3, was analysed. It would further be desirable for such studies to have objective data, in particular to obtain a valid event exposure measure that is not retrospectively assessed two years after the event, as in the present data. Assessments of subsequent exposure to major life events would be desirable because such experience could be responsible for symptoms at a later point in time. Particularly in police officers, responses to traumas in the community that may involve potential violence, this could be evaluated by analyses of the police officers’ responses to such events.

In addition, baseline data of emotional support would have been valuable to control for pre-existing individual differences. The question whether support predicts mental health conditions or rather vice versa has been discussed for several decades with the conclusion that there are reciprocal effects but the mechanisms differ between samples, time points, and type of traumatic events. For example, the social support deterioration model [25] states that post-disaster declines in support mediate the impact of disaster stress. The same authors found two contrasting causal mechanisms over time in a different study where more social support led to less PTSD in the earlier post-disaster phase, 6 to 12 months after the impact, whereas more PTSD led to less social support at 18 to 24 months after the event [26]. Cross-lagged panel analyses for PTSD and social relationship satisfaction yielded inconsistent results although there was a predominant path from satisfaction to natural recovery [27]. In veterans of the Gulf war, the directionality of the association between social support and PTSD suggested that interpersonal problems associated with PTSD may have a detrimental effect on social support, as there was a strong negative relationship between PTSD at Time 1 with support at Time 2, whereas support at Time 1 did not predict PTSD at Time 2 [28]. Findings like this one suggest that it is worthwhile to study social support also as an outcome variable in stressful times.

Moreover, self-reports of stress levels by police officers may beunder-reported. The police likely represent a unique culture compared to area residents in their reluctance to reveal and self-disclose due to fears of job consequences which may result, for example, in losing the privilege of carrying a weapon [29]. Police are generally seen as a highly resilient group due to their selection into their jobs and due to training and experience. In an analysis of police officers following exposure to a life-threatening event, three discrete PTSD symptom trajectories were revealed, namely resilient, distressed–improving, and distressed–worsening [30]. In another study, active-duty officers were assessed four times over four years, and four trajectories with different levels of symptomatology were found, indicating that resilience is predicted by emotions prior to stressor exposure [31]. This underscores the need to have baseline data to gauge the mechanisms that are involved in long-term changes in PTSD.

Moreover, such studies also point to the advantage of going beyond a single trajectory, using latent growth mixture models that permit uncovering unknown subgroups in the sample for which there might be different trajectories. In the present analyses, the focus has been on the mediator chain from exposure via PTSD to emotional support, and therefore it was straightforward to assume a single trajectory, not extending the research question by focussing on possible heterogeneity of the sample. However, future analyses with the aim to disentangle different intercepts and slopes for subgroups of police officers might benefit from advanced methods that allow to further explore differential pathways. In spite of the high potential that such methods offer, they also have their limitations such as the justification for the number of latent classes and their interpretation.

## Conclusions

In conclusion, this three-wave reanalysis of the WTCHR data of police responders with a narrow focus on exposure, PTSD symptoms, and emotional support, adds a different view to the large number of other research reports on this sample that have chosen a broader scope (e.g., [8]). To model the longitudinal PTSD symptoms as one latent trajectory comprising three waves of data collection yielded findings that shed light on the stability of exposure effects, and address the issue of emotional support loss for those suffering from symptoms over time. Future longitudinal post-disaster studies should assess various types and sources of social support - in their initial evaluation to account for changes in emotional and instrumental support receipt and provision across time. Moreover, unknown subgroups of this population could be examined instead of the present focus on one single PTSD trajectory.

## Abbreviations

CFI, comparative fit index; PCL, PTSD Checklist; PTSD, posttraumatic stress disorder; SRMR, standardized root mean square residual; WTC, World Trade Center; WTCHP, World Trade Center Health Program
